# Revision and reannotation of the *Halomonas elongata* DSM 2581^T^ genome

**DOI:** 10.1002/mbo3.465

**Published:** 2017-03-27

**Authors:** Friedhelm Pfeiffer, Irina Bagyan, Gabriela Alfaro‐Espinoza, Maria‐A. Zamora‐Lagos, Bianca Habermann, Alberto Marin‐Sanguino, Dieter Oesterhelt, Hans J. Kunte

**Affiliations:** ^1^ Computational Biology Group Max‐Planck‐Institute of Biochemistry Martinsried Germany; ^2^ Research and Development Division bitop AG Witten Germany; ^3^ Materials and Environment Division Federal Institute for Materials Research and Testing (BAM) Berlin Germany; ^4^ Faculty of Mechanical Engineering Specialty Division for Systems Biotechnology Technische Universität München Germany; ^5^ Department of Membrane Biochemistry Max‐Planck‐Institute of Biochemistry Martinsried Germany

**Keywords:** frameshift, genome annotation, genome sequencing, Halomonas elongata, halophilic bacteria, sequence revision

## Abstract

The genome of the *Halomonas elongata* type strain DSM 2581, an industrial producer, was reevaluated using the Illumina HiSeq2500 technology. To resolve duplication‐associated ambiguities, PCR products were generated and sequenced. Outside of duplications, 72 sequence corrections were required, of which 24 were point mutations and 48 were indels of one or few bases. Most of these were associated with polynucleotide stretches (poly‐T stretch overestimated in 19 cases, poly‐C underestimated in 15 cases). These problems may be attributed to using 454 technology for original genome sequencing. On average, the original genome sequence had only one error in 56 kb. There were 23 frameshift error corrections in the 29 protein‐coding genes affected by sequence revision. The genome has been subjected to major reannotation in order to substantially increase the annotation quality.

## 1 Introduction

*Halomonas elongata* is an aerobic and halophilic microorganism that belongs to the γ‐proteobacteria. *H. elongata* can thrive at low and high salinity. The major osmoregulatory mechanism that allows for growth in saline environments is the ability of *H. elongata* to accumulate the compatible solute ectoine (Ventosa, Nieto, & Oren, 1998). Ectoine is amassed inside the cytoplasm either by de novo synthesis via aspartate (Peters, Galinski, & Trüper, 1990; Schwibbert et al., 2011) or by uptake from the surrounding environment with the help of the ectoine‐specific, osmoregulated transporter TeaABC (Grammann, Volke, & Kunte, 2002; Kuhlmann, Terwisscha van Scheltinga, Bienert, Kunte, & Ziegler, 2008). *H. elongata* can grow on a variety of carbon sources (Vreeland, Litchfield, Martin, & Elliot, 1980) and even utilizes ectoine as sole carbon and/or nitrogen source, a capability restricted to only few ectoine‐producing bacteria (Schwibbert et al., 2011).

We have reported the complete genome sequence of *H. elongata* in 2011 (Schwibbert et al., 2011). Although the number of disrupted genes was small, we subsequently found several of them to be functional. Resequencing of individual genes identified frameshift errors in the original 454‐based genome sequence. Here, we report an improved genome sequence based on a large‐scale resequencing effort using Illumina technology.

## 2 Materials and Methods

### 2.1 Genome resequencing and genome error identification

Having identified several functional protein‐coding genes, which seemed disrupted according to our genome sequence, we decided to resequence the genome using the Illumina HiSeq2500 technology (GATC Biotech, Konstanz, Germany) for a high genome coverage. The reads were used to pinpoint genome sequence errors, applying the methods described for an *Aeromonas salmonicida* genome sequencing project (Zamora‐Lagos et al., in prep.). In brief, reads were mapped to the reference genome (using BowTie, TopHat), followed by mismatch and indel detection (with Samtools, Varscan) (Koboldt et al., 2009; Langmead & Salzberg, 2012; Li et al., 2009; Trapnell, Pachter, & Salzberg, 2009). In the *A. salmonicida* genome project, we had randomly spotted additional sequence errors, which had not been detected by the above tools. Therefore, we developed a k‐mer‐based algorithm that permitted to identify additional sequence errors (Zamora‐Lagos et al., in prep.). Briefly, k‐mers (49‐mers) were extracted from the Illumina reads, applying a one‐step window. For each k‐mer, the occurrence count in the Illumina reads was determined (with k‐mers and their reverse complements being merged). In parallel, k‐mers were identified in the genome sequence. At each of the bases covered by a k‐mer, the occurrence count was added in order to estimate Illumina read coverage. The resulting data were analyzed in two ways: (a) We searched for drops in Illumina read coverage, which are expected to indicate genome sequence errors. The corresponding genome positions were subjected to manual curation. (b) Also, we searched for k‐mers with a high occurrence count in the Illumina reads, which are not found in the genome sequence. An attempt was made to identify the genome region to which these reads belong. In several cases, this uncovered additional polymorphic versions of duplicated genome regions.

### 2.2 Genome reannotation

In the original genome sequencing report, we had only subjected the metabolic network relevant for ectoine biosynthesis to detailed manual curation. Thereafter, we have improved the annotation by applying manual curation to the complete genome. (a) For about one‐third of the genome, we applied a stringent annotation strategy (Pfeiffer & Oesterhelt, 2015). This strategy requires that annotations are transferred only from homologs, which themselves have been subjected to experimental analysis (Gold Standard Proteins). Briefly, we use the SwissProt section of UniProt as a rich source for such proteins (last accessed release: 2017_01, 18‐Jan‐2017) (UniProt Consortium, 2016). In addition, a significant amount of literature work has been performed during this project. Literature work was mainly required when experimental data were yet unavailable via UniProt (we provided feedback to UniProt in such cases). (b) As this procedure is extremely time‐consuming, we developed a more relaxed annotation strategy for the remainder of the genome. This is based on our experience that annotations in the SwissProt section of UniProt are highly reliable. Proteins were compared to the SwissProt section of UniProt using BLASTp (Altschul et al., 1997). Homologs with high levels of sequence identity (seqid) (commonly >50%) were assumed to be equivalogs (orthologs with identical function). The SwissProt annotation was transferred to the *H. elongata* protein‐coding genes. In case of more distantly related homologs (40%–50% seqid), the SwissProt sequence was compared to the *H. elongata* proteins using BLASTp to verify sequence orthology before annotation transfer. For proteins with only more distant homologs in SwissProt functional conservation was considered unlikely. Thus, only a general protein name was assigned in order to escape the “overannotation problem” (Pfeiffer & Oesterhelt, 2015). In such cases, protein names are shaped according to the more distant UniProt/SwissProt homologs and/or assigned InterPro domains in order to keep a high level of naming consistency. We also attempted to minimize EC number assignment inconsistencies between our annotation and the KEGG database (last accessed: 17‐Jan‐2017) (Kanehisa, Furumichi, Tanabe, Sato, & Morishima, 2017).

## 3 Results and Discussion

The available genome sequence is mainly based on data obtained with the 454 technology (Schwibbert et al., 2011). Upon further study of the organism, we found several genes, which were expected to be functional but were disrupted by frameshift according to the genome sequence. Examples are PyrC (Helo_2340), required for pyrimidine biosynthesis and MoeA (Helo_2621), required for MoCo biosynthesis. Also, Zwf (Helo_3637) is required for the cytosolic conversion of glucose‐6‐phosphate to gluconate‐6‐phosphate at the beginning of the Entner‐Doudoroff pathway. Low‐scale resequencing of PCR products showed that several of these genes were not disrupted by frameshift but that the genome sequence was not correct. Therefore, we decided for a systematic approach to increase genome sequence reliability. We selected the Illumina HiSeq2500 technology and achieved a 650‐fold genome coverage (21 million reads of 2.6 Gbp total length).

Initially, we applied standard technologies for detection of differences between the reference genome and the set of Illumina reads (read mapping, followed by mismatch and indel detection). Commonly, such tools are used for SNP (single‐nucleotide polymorphism) analysis. However, a sequence error in the reference genome can be considered as an equivalent to a mutant in a sequence dataset. By this approach, we identified 62 genome sequence errors. We had very recently performed another such analysis in a genome sequencing project on *Aeromonas salmonicida subsp. pectinolytica* 34mel (Zamora‐Lagos et al., in prep.). During that project, we had randomly spotted genome sequence errors which had been overlooked by the standard methodology. Several of the corresponding errors proved to be unusually severe (e.g., three closely spaced point mutations), which interfered with read mapping at the applied stringency. Thus, the genome sequence differed considerably from that of the Illumina reads but the standard methodology did not list this case. As a consequence, a required correction of a more severe genome error would have been missed while the minor errors would have been corrected.

To overcome this problem, we developed a complementary parameter‐independent algorithm for detection of potential genome errors (Zamora‐Lagos et al., in prep.). Upon application of this methodology to the *H. elongata* genome, we detected only very few additional problems in unique genome regions. However, our algorithm proved efficient to identify previously overlooked genome sequence errors in duplicated regions of the *H. elongata* genome.

(1) In one case, two copies of a gene (Helo_1778 and Helo_2803) were previously considered truly identical over the N‐terminal 335 codons, even at the DNA sequence level, while our analysis uncovered that the sequences differ at two base positions, both mutations being silent. This problem escaped detection by the standard methodology (reads for the incorrect sequence are available due to the other gene copy and are mapped to both genome regions, thus masking the problem). However, as the variant sequences were frequent in the Illumina reads but were missing in the genome, they were detected as high‐frequency novel k‐mers. (2) In the second case, the sequence of the transketolase gene *tktA2* (Helo_1184) had to be revised at two sites. Previously, this had been considered identical to *tktA1* (Helo_3966) at the nucleotide sequence level. At one site, there was a trinucleotide difference, the other being a point mutation only 7 bp apart. Both mutations are silent (Table 1). The trinucleotide difference switches a TCG codon for Ser into AGC. This problem escaped detection by the standard methodology due to the applied high mapping stringency. (3) The third case affected gene *vgr2* (Helo_2928), coding for the T6SS‐related Vgr family protein. This was previously considered identical to *vgr3* (Helo_4077) for the N‐terminal 643 codons. However, there are 23 point mutations as revealed by a manual polymorphism linkage analysis. This result was confirmed by PCR amplification with primers from unique adjacent genome regions and subsequent sequencing. The mutations result in five amino acid changes. Compared to the remainder of the genome, this is a 1,000‐fold higher error density and we tried to identify what had caused this problem. It turned out that the originally reported *vgr2* gene had been read from a gap‐closing PCR product. This product had been generated by primers which turned out to be very far apart in the final genome sequence, namely one from the *vgr2* region and the other from the *vgr3* region. Most likely, the generation of this PCR product involved correct priming by the *vgr3*‐related primer and mispriming by the *vgr2*‐related primer. Due to the high sequence similarity of the *vgr2* and *vgr3* gene sequences (97% nucleotide sequence identity), this problem went undetected. (4) The fourth case is a genome region with several tandem copies of a 73‐mer sequence with polymorphisms at three internal positions. Of the eight theoretically possible sequence versions, five were identified in the Illumina read set at high frequency, only four of which are contained in the original genome sequence. Upon PCR amplification and sequencing, we could not only position the fifth polymorphic variant but we also realized that there are not 8 but 15 copies of the 73‐mer, leading to a 511 bp insertion.

**Table 1:** List of proteins affected by genome sequence error corrections

Code	Mutation class	Gene	Protein name
Helo_1184	Silent mutation	tktA2	Transketolase
Helo_1373	Protein sequence differs	–	Dodecin domain protein
Helo_1605	Repair of known frameshift	pykA2	Pyruvate kinase
Helo_1778	Silent mutation	–	TRAP transporter large transmembrane protein
Helo_1905	Repair of known frameshift	puuC1	Aldehyde dehydrogenase PuuC
Helo_1959	Repair of known frameshift	murD	UDP‐N‐acetylmuramoylalanine–D‐glutamate ligase
Helo_2138	C‐term region replaced	–	ABC‐type transport system ATP‐binding protein
Helo_2340	Repair of known frameshift	pyrC	Dihydroorotase
Helo_2343	Repair of known frameshift	luxS	S‐ribosylhomocysteine lyase
Helo_2397H	Repair of known frameshift	–	Conserved hypothetical protein
Helo_2621	C‐term region replaced	moeA	Molybdopterin molybdenumtransferase
Helo_2736	Repair of known frameshift	–	CstA family protein
Helo_2780	C‐term region replaced	–	Glycoside hydrolase family protein
Helo_2822	Silent mutation	–	Aldolase domain protein
Helo_2823	Silent mutation	–	DapA domain protein
Helo_2823A	Repair of known frameshift	–	Conserved hypothetical protein
Helo_2928	Protein sequence differs	vgr2	T6SS‐related Vgr family protein
Helo_2941H	Repair of known frameshift	–	DUF867 family protein
Helo_3063A	Repair of known frameshift	rluE	Ribosomal large subunit pseudouridine synthase RluE
Helo_3106	C‐term region replaced	–	Glycosyltransferase domain protein
Helo_3291A	Repair of known frameshift	–	DUF2971 domain protein
Helo_3428	Repair of known frameshift	slt	Lytic murein transglycosylase Slt
Helo_3567	N‐term region replaced	plsB	Glycerol‐3‐phosphate acyltransferase
Helo_3606	C‐term region replaced	–	ABC‐type transport system ATP‐binding protein
Helo_3637	Repair of known frameshift	zwf	Glucose‐6‐phosphate 1‐dehydrogenase
Helo_3927	C‐term region replaced	–	Glycoside hydrolase domain protein
Helo_4206	C‐term region replaced		Probable methyltransferase (homolog to DNA‐cytosine methyltransferase)
Helo_4313	N‐term region replaced	–	NSS family transport protein
Helo_4398B	N‐term region replaced	–	Conserved hypothetical protein

Within unique genome regions, we found 72 differences (Table 2), with fewer simple mutations (23 out of 24 were point mutations) than frameshifts (48). There were only three simple one‐base frameshifts. Most frameshifts (37) occurred in polynucleotide runs. In 19 cases, T‐runs were one base too long; in 15 cases, C‐runs were one base too short; in the remaining three cases, C‐runs were one base too long. The overrepresentation of homopolymer‐related frameshifts in a sequence originally obtained by 454 pyrosequencing is noteworthy.

**Table 2:** Summary of genome sequence error corrections

Genome category	Mutation category	Mutation class	Number	Sum1	Sum2	Total
Unique region	Nonshifting	Point mutation	23	24	72	100
		Trinucleotide mutation	1			
	Indel	Simple one‐base indel	3	48		
		A/T polymer one base too long	19			
		C/G polymer one base too long	3			
		C/G polymer one base too short	15			
		Other frameshift	8			
Duplication	Nonshifting	Point mutation	26	27	28	
		Trinucleotide mutation	1			
	Indel	Long indel (511 bp)	1	1		

Differences are summarized based on genome category (duplication‐associated or unique), mutations category (nonshifting or indel). Numbers of cases are provided for different mutation classes and summarized by mutation category (sum1), by genome category (sum2), and for all cases (total).

Overall, 29 protein‐coding genes were affected by mutations (Table 1, Table 3). Of the six genes affected only by point mutation, two have a modified protein sequence, while the mutations are silent in the other four. There were 23 frameshift differences. In 13 cases, the gene was annotated as disrupted but is functional. In the other 10 cases, the gene was annotated as functional but had an invalid C‐terminal (7) or N‐terminal (3) sequence.

**Table 3:** Protein‐coding genes affected by genome sequence error corrections

Mutation category	Sequence revision class	Number	Sum	Total
Nonshifting mutation only	Silent mutation	4	6	29
	Protein sequence differs	2		
Frameshift	Repair of known frameshift	13	23	
	C‐term region replaced	7		
	N‐term region replaced	3		

Numbers of cases are provided for different sequence revision class and summarized for mutation categories and for all cases (total).

In the original sequencing report, we applied a stringent annotation strategy only to proteins directly connected to ectoine metabolism, while the remainder of the genome was left as predicted by an annotation robot (Schwibbert et al., 2011). In the meantime, we have applied the stringent annotation strategy (Pfeiffer & Oesterhelt, 2015) to one‐third of the genome. This detailed postannotation was performed during a study concerned with detailed analysis of osmoregulation in *H. elongata* (Kindzierski et al., 2017). For the remainder, a more relaxed manual curation strategy was applied which benefits from the high reliability of annotations in the UniProt/SwissProt database (see methods). For a significant number of genes, start codons were reassigned, applying a BLAST‐based start codon checking procedure (Pfeiffer et al., 2008). Also, a number of previously missed gene annotations were resolved by postprediction, commonly detected in long “intergenic” regions. The genome reannotation was managed in HaloLex (Pfeiffer et al., 2008). The revised and reannotated genome has a length of 4,061,825 bp (previously 4,061,296 bp) and has 3,644 protein‐coding genes.

Some aspects of gene reannotation are illustrated by the genome region from 1.028 to 1.054 Mb, covering 21 genes (Helo_1886 to Helo_1906). The region contains proteins of polyamine metabolism related to both, the *Escherichia coli puuPABCDR* and the *Pseudomonas aeruginosa spuABCDEFGHI* cluster. Helo_1905 is one of the proteins with sequence correction, resolving a frameshift error in the original genome sequence. There is a very close paralog (Helo_3918) with 97% protein sequence identity (seqid) to Helo_1905 and 53% seqid to *E. coli* PuuC, an aldehyde dehydrogenase with a broad substrate specificity. Helo_1905 is annotated as puuC1 due to its genetic neighborhood with homologs to *E. coli* PuuA (Helo_1902), PuuB (Helo_1893), PuuD (Helo_1903), and PuuR (Helo_1906). Adjacent to PuuC1 is Helo_1904, a class‐III aminotransferase which had *spuC* as initially assigned gene symbol. This assignment seemed to lack sufficient support and was initially rejected. The best UniProt/SwissProt homolog is DoeD from *H. elongata* with 46% seqid. However, a literature search identified a paper describing the *P. aeruginosa spuABCDEFGHI* cluster (Lu, Itoh, Nakada, & Jiang, 2002), which has not yet been processed by UniProt (they were made aware of this paper by feedback). SpuC (putrescine aminotransferase) shows 65% seqid to Helo_1904 and up to 43% seqid to other class‐III aminotransferases. A homolog of SpuB (Helo_1891) is located adjacent to homologs of the SpuDEFGH spermidine/putrescine ABC transporter (*spuFGHE1*: Helo_1887 to Helo_1890, *spuE2*: Helo_1892). Both clusters contain two periplasmic substrate‐binding proteins which all are equidistant to each other (about 32% seqid), but without a clear one‐to‐one correspondence. In *P. aeruginosa*, SpuD is responsible for putrescine transport and SpuE, for spermidine transport (Wu et al., 2012). As the substrate specificity cannot be securely predicted for the *H. elongata* homologs, we assigned gene names *spuE1* and *spuE2*. *P. aeruginosa* SpuA is somewhat more closely related to Helo_1903 than *E. coli* PuuD (49% and 44% seqid, respectively) but only *E. coli* PuuD has been functionally characterized and thus this annotation is preferred for the *H. elongata* homolog.

Finally, we want to comment on one annotation detail, the 3′ end of the 16S rRNA. We consider GGATCACCTCCTTA to be the correct 3′ sequence, while our previous annotation had a 2 nt extension (AT). Our previous annotation was based on the publication by Lin, Chang, Chiang, & Tang (2008). These authors are clearly correct in their general conclusion that many 16S rRNA sequences in the databases are too short because they lack the conserved anti‐SD sequence CCTCC. However, the authors added 5 instead of 3 nt after the anti‐SD sequence, even for the *E.coli* 16S rRNA (Suppl.Table S1 page 36). The additional dinucleotide (CC) is inconsistent with the 3′ end of E.coli 16S rRNA (ACCTCCTTA) as experimentally determined by Shine & Dalgarno, (1974). Our previous setting of the 16S rRNA 3′ end was consistent with Lin et al., while the revised 3′ end is consistent with Shine and Dalgarno. The corrected 3′ end 16S rRNA is consistent with the annotation in RFAM (last accessed: 5‐Dec‐2016) (Nawrocki et al., 2015). We also made other RNA annotations consistent with RFAM except for tRNAs which we made consistent with GtRNADB (Chan & Lowe, 2016).

The nucleotide sequence accession number is FN869568, sequence version 2.

## Conflict of Interest

The authors declare no conflict of interest.
